# Comparative Study Between the First and Second Wave of COVID-19 Deaths in India: A Single Center Study

**DOI:** 10.7759/cureus.37472

**Published:** 2023-04-12

**Authors:** Prakash Tendulkar, Pragya Pandey, Prasan K Panda, Ajeet S Bhadoria, Poorvi Kulshreshtha, Mayank Mishra, Gaurika Saxena

**Affiliations:** 1 Internal Medicine, All India Institute of Medical Sciences, Rishikesh, Rishikesh, IND; 2 Community and Family Medicine, All India Institute of Medical Sciences, Rishikesh, Rishikesh, IND; 3 Physiology, All India Institute of Medical Sciences, Rishikesh, Rishikesh, IND; 4 Epidemiology and Public Health, All India Institute of Medical Sciences, Rishikesh, Rishikesh, IND

**Keywords:** risk factors, pandemic waves, mortality, coronavirus disease 2019, comorbidities

## Abstract

Introduction

The severe acute respiratory syndrome coronavirus 2 (SARS-CoV-2) is continuously evolving, and many mutant variants of the virus are circulating in the world. Recurrent waves of COVID-19 have caused enormous mortality all across the globe. Considering the novelty of the virus, it becomes crucial for healthcare experts and policymakers to understand the demographic and clinical attributes of inpatient deaths in the first and second waves of COVID-19.

Methods

This hospital record-based comparative study was conducted at a tertiary care hospital in Uttarakhand, India. The study included all COVID-19 RT PCR-positive patients admitted to the hospital during the first wave, from 1^st^ April 2020 to 31^st^ January 2021, and the second wave from 1^st^ March 2021 to 30^th^ June 2021. Comparisons were made with respect to demographic, clinical, laboratory parameters, and course of hospital stay.

Results

The study exhibited 11.34% more casualties in the second wave, with the number of deaths being 424 and 475 for the first and second waves, respectively. A male preponderance of mortality was evident in both waves with significant differences (p=0.004). There was no significant difference in age between the two waves (p=0.809). The significantly different comorbidities were hypertension (p=0.003) and coronary artery disease (p=0.014). The clinical manifestations demonstrating a significant difference were cough (p=0.000), sore throat (p=0.002), altered mental status (p=0.002), headache (p=0.025), loss of taste and smell (p=0.001), and tachypnea (p=0.000). The lab parameters with a significant difference across both waves were lymphopenia (p=0.000), elevated aspartate aminotransferase (p=0.004), leukocytosis (p=0.008), and thrombocytopenia (p=0.004). During the hospital course of the second wave, in terms of intensive care unit stay, the need for non-invasive ventilation and inotrope support was higher. The complications manifesting in the form of acute respiratory distress syndrome and sepsis were observed more in the second wave. A significant difference was discerned in the median duration of hospital stay in both waves (p=0.000).

Conclusion

Despite being of shorter duration, the second wave of COVID-19 culminated in more deaths. The study demonstrated that most of the baseline demographic and clinical characteristics attributed to mortality were more common during the second wave of COVID-19, including lab parameters, complications, and duration of hospital stays. The unpredictable nature of COVID-19 waves calls for instituting a well-planned surveillance mechanism in place to identify the surge in cases at the earliest possible time and prompt response, along with developing infrastructure and capacity to manage complications.

## Introduction

The emergence and rapid spread of coronavirus disease 2019 (COVID-19) have caused enormous mortality worldwide. This pandemic witnessed massive deaths, which the world has not seen in the last 100 years. The World Health Organization (WHO) declared COVID-19 a global pandemic on March 11, 2020. Since then, India has witnessed three waves. Compared to the first wave, the second wave has had severe consequences worldwide regarding cases and mortality. Even in India, mortality was higher in the second wave than in the first wave [1]. As of March 11, 2023, more than 446 million RT-PCR-confirmed cases had been diagnosed in India, with more than 5.3 lakh deaths attributed to the COVID-19 infection [2].

The first wave had a prolonged course from March 2020 to approximately January 2021. In contrast, the second wave had a rapid course that started in March-April 2021, and over the next 1-2 months, there was a sharp rise in cases and related deaths [3]. This slowness in the first wave and suddenness in the second wave may have impacted morbidity and mortality.

A descriptive study is already done at the same center to assess the first wave's comorbidities, risk factors, clinical signs and symptoms, and other baseline characteristics [4]. The present study is aimed at looking for differences in baseline characteristics between the first and second waves at a tertiary care hospital in Uttarakhand, India.

## Materials and methods

Study design

A hospital record-based comparative study.

Study setting

All India Institute of Medical Sciences, Rishikesh, is a tertiary care hospital in Uttarakhand, India. 

Study duration

The first wave from 1st April 2020 to 31st January 2021, and the second wave will run from 1st March 2021 to 30th June 2021.

Inclusion and exclusion criteria

The study included all COVID-19 RT-PCR-positive patients admitted to the hospital. COVID-19 RT-PCR-positive patients who were declared dead at the hospital were excluded from the study. 

Study methodology

All the patients who tested positive for COVID-19 on RT-PCR were admitted to the designated COVID ward of the hospital, and their demographic, baseline clinical, and laboratory parameters were recorded at the time of stay. Their clinical progress note was updated with respect to the need for an intensive care unit (ICU), non-invasive ventilation (NIV), inotrope support, and renal replacement therapy (RRT), and the outcome was also updated either in the form of a discharge note or a death certificate on the hospital health management information system (HMIS) portal authorized by the government of India. Data were retrieved from the HMIS portal after obtaining appropriate ethical approval from the institute’s ethical committee (CTRI/2020/08/027169). 

Statistical analysis

Comparisons were made with respect to demographic, clinical, laboratory parameters, and course of hospital stay. The retrieved data was duly managed in a Microsoft Excel (Redmond, USA) spreadsheet. Data analysis was conducted on IBM Corp. Released 2017. IBM SPSS Statistics for Windows, Version 25.0. Armonk, NY: IBM Corp. As part of descriptive statistics, categorical variables are reported as frequencies and proportions, the interval between symptom onset and hospital admission and the duration of hospital stay is expressed as the median with an interquartile range, and other continuous variables are reported as the mean ± standard deviation. The proportions and median were compared by applying the Chi-square test and Mann-Whitney U test, respectively.

## Results

**Figure 1:**
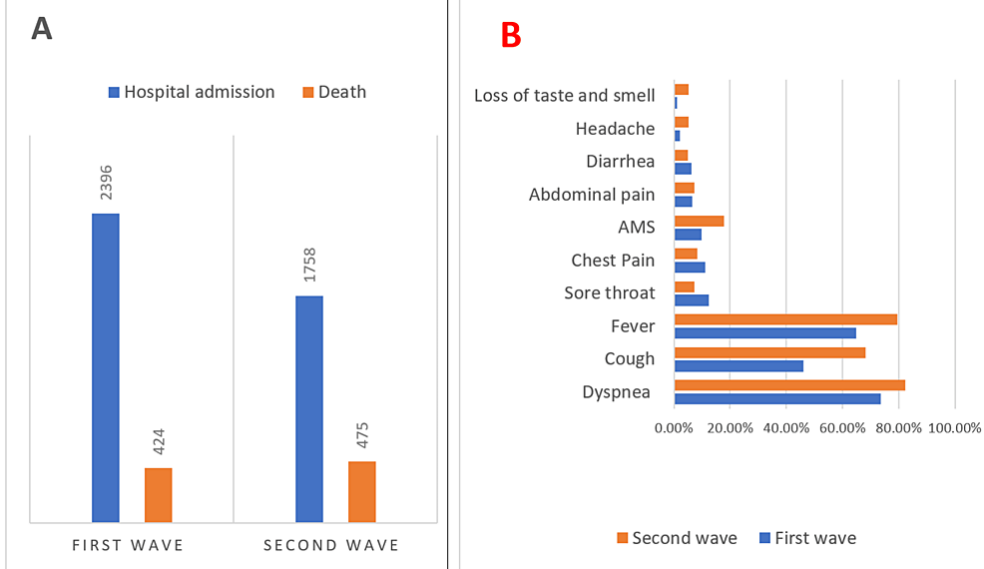
Baseline characteristics of COVID-19 deaths during the first and second waves, with total admission and death (A) and symptom distribution (B).

Out of the total reported COVID-19 cases till January 2021 (the first wave), 2396 were admitted to this tertiary care hospital, and 424 causalities were reported. During the second wave of COVID-19, a total of 1758 patients were admitted, out of whom 475 casualties were reported (Figure 1A).

The baseline characteristics of the patients are shown in Table 1. In the first wave, the mean age of the patients was 55 ± 16.24 years (95 % CI, 54.35-57.45), while in the second wave, the mean age in years was 56.81 ± 14.92 years (95 % CI, 55.46-58.15). The mean age was not statistically different among both groups (p=0.384). Most of the deceased in both waves were older than 45 years, with no statistical difference among the young, middle, and elderly age groups. There was a significant difference in both waves with respect to gender (p<0.004). The male preponderance of mortality was evident in both waves. The median number of days between the onset of symptoms and hospital admission was six (3-11) days in the second wave, which is significantly different from the corresponding interval in the first wave (p<0.001). The median duration of hospital stay was five (3-10) days in the second wave, which is significantly different from the corresponding time in the first wave (p<0.001).

**Table 1:** Baseline characteristics (proportions in percentages with exact numbers) of patients in the first and second COVID-19 waves AMS: Altered mental status, DM: Diabetes mellitus, HTN: Hypertension, CAD: Coronary artery disease, COPD: Chronic obstructive pulmonary disease, CKD: Chronic kidney disease, CLD: Chronic liver disease, CND: Chronic neurological disease, ALT: Alanine aminotransferase, AST: Aspartate aminotransferase, AKI: Acute kidney injury, NIV: Non-invasive ventilation, ARDS: Acute respiratory distress syndrome

Variable		First wave (N-424)	Second wave (N-475)	P -Value
Gender	Male	70.3% (298)	61.3% (291)	0.004
	Female	29.7% (126)	38.7% (184)	
Age	<45 years	20.51% (87)	21.1% (100)	0.809
	45-60 years	35.37% (150)	35.2% (168)	
	>60years	44.10% (187)	43.6% (207)	
Symptoms	Dyspnea	73.6% (312)	82.3% (391)	0.074
	Cough	46.1% (196)	68.2% (324)	<0.001
	Fever	64.92% (275)	79.4% (377)	0.228
	Sore throat	12.5% (53)	7.2% (34)	0.002
	Chest Pain	11.2% (48)	8.4% (40)	0.183
	AMS	9.8% (42)	17.9% (85)	0.002
	Abdominal pain	6.5% (28)	7.4% (35)	0.668
	Diarrhea	6.3% (26)	5.1% (24)	0.311
	Headache	2.2% (9)	5.3% (25)	0.025
	Loss of taste and smell	1.1% (5)	5.3% (25)	0.001
Sign	Tachypnea	80% (339)	94.9% (451)	<0.001
	Hypoxia	64.86% (275)	71.10% (338)	0.061
	Hypotension	8.1% (35)	9.47% (45)	0.06
Comorbidity	DM	41.4% (176)	42.5% (202)	0.745
	HTN	39.8% (169)	31.6% (150)	0.003
	CAD	15.2% (65)	10.7% (51)	0.014
	COPD	8.3% (35)	6.9% (33)	0.310
	CKD	7.6% (32)	8.4% (40)	0.843
	CLD	3.4% (15)	5.5% (26)	0.118
	Malignancy	5.4% (23)	3.4% (16)	0.127
	CND	3.6% (15)	5.9% (28)	0.102
Lab Parameters	Lymphopenia	90.2% (383)	86.1% (409)	<0.001
	Raised ALT	59.7% (253)	62.3% (296)	0.468
	Raised AST	35.1% (149)	44.8% (213)	0.004
	Leukocytosis	50.5% (214)	60.4% (287)	0.008
	AKI	37.7% (160)	41.5% (197)	0.266
	Anemia	28.8% (122)	28.2% (134)	0.799
	Thrombocytopenia	25.1% (106)	35.2% (167)	0.004
Hospital course	ICU stay	78.3% (332)	94.5% (449)	<0.001
	NIV Need	55.9% (237)	85.5% (406)	<0.001
	Inotropes support	54.7% (232)	62.9% (299)	0.010
	Renal replacement therapy	10.3% (44)	4.4% (21)	<0.001
Complications	ARDS	68.87% (292)	87.15% (414)	<0.001
	Sepsis	40.33% (171)	65.05% (309)	<0.001

The most common clinical manifestation among the deceased was dyspnea in both waves, followed by fever and cough. Other less common symptoms were a sore throat, chest pain, altered mental status, abdominal pain, diarrhea, headache, and loss of taste and smell. Symptoms in the form of cough, sore throat, altered mental status, and loss of taste and smell were more common among the deceased in the second wave, which was statistically significant (p<0.05) (Figure 1B).

**Figure 2:**
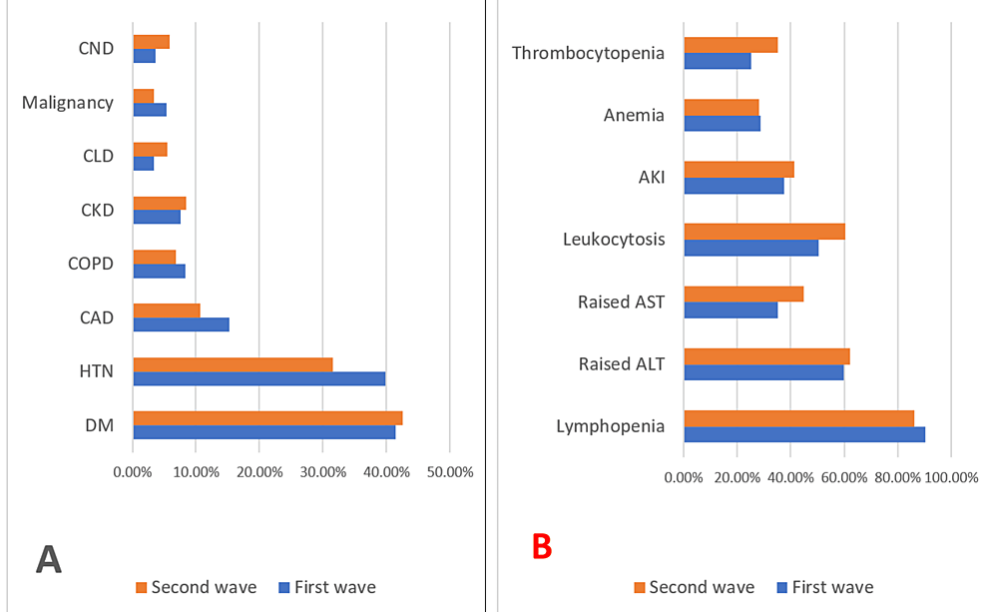
Baseline characteristics of COVID-19 deaths during the first and second waves with comorbidities (A) and deranged laboratory parameters (B). DM: Diabetes mellitus, HTN: Hypertension, CAD: Coronary artery disease, COPD: Chronic obstructive pulmonary disease, CKD: Chronic kidney disease, CLD: Chronic liver disease, CND: Chronic neurological disease, ALT: Alanine aminotransferase, AST: Aspartate aminotransferase, AKI: Acute kidney injury

The most prevalent comorbidity among the deceased in both waves was diabetes mellitus, followed by hypertension. Other less common comorbidities were coronary artery disease, chronic obstructive pulmonary disease, chronic kidney disease, chronic liver disease, chronic neurological disease, and solid and hematological malignancies. There was a significant difference in both waves with respect to hypertension and coronary artery disease. Both medical comorbidities were more prevalent in the first wave (Figure 2A).

The most frequently deranged lab parameter was lymphopenia among the deceased in the first and second waves. Other derangements in lab investigations were raised alanine aminotransferase, aspartate aminotransferase, and leucocytosis. Acute kidney injury, anemia, and thrombocytopenia were widespread among deceased patients. There was a statistically significant difference in both waves with respect to lymphopenia, thrombocytopenia, raised aspartate aminotransferase, and leucocytosis (p<0.05) (Figure 2B).

In-hospital, of course, most patients in the second wave needed ICU, NIV, and inotropic support. During the second wave, complications like acute respiratory distress syndrome (ARDS) and sepsis were common.

## Discussion

**Figure 3:**
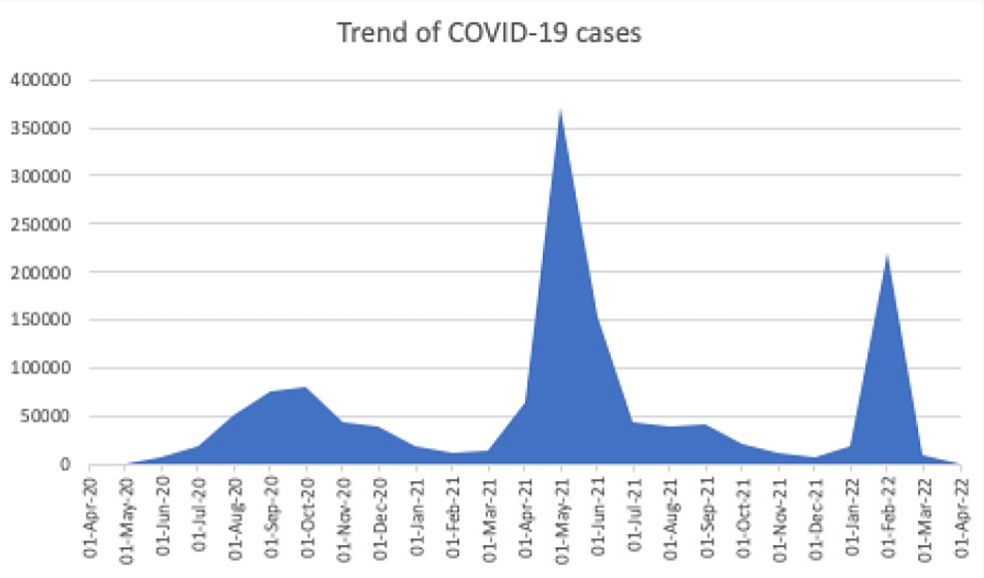
Trend of COVID-19 cases in India (authors prepared from available open-source data).

India witnessed the first wave of COVID-19 in 2020, which peaked in September 2020, and gradually the cases declined. Again, in March 2021, new infections with the latest virus variant began to tick up, and over the next 3-4 months, the country saw medical chaos, a falling health infrastructure, and deaths in large numbers. Experts say the official death toll is even higher than the official data suggest (Figure 3) [5]. We did this retrospective study to compare some baseline characteristics between the first and second waves of COVID-19.

In this study, the mean age of the patients in the first and second waves was very similar at 55 and 56, respectively. A similar mean age group was seen in previous studies and systematic reviews; in one of the systematic reviews, consisting of 32 studies, the mean age was 56 years [6]. In a similar review on critical patients, the mean duration of ICU stays for critical patients was nine days [6]. In one of the studies done in Pune, India, death from the diagnosis took six days [7]. In this study, we also had a similar duration of hospital stay in both waves. The median duration of hospital stay in the second wave was five days, while in the first wave, it was nine days. The significant difference in the duration of hospital stay between the first and second waves indicates the relentless severity of the disease during the second wave.

Older age is an independent factor for increased mortality among patients, and several previous studies among ICU patients have shown a similar trend [8,9]. Previous research also suggests an age-dependent defect in T-cell and B-cell functions; also, excess production of type 2 cytokines can lead to a prolonged proinflammatory response and insufficiency in preventing viral replication, leading to poor outcomes [10]. In this study, most patients were older than 45 (~80%).

Most previous studies have shown a male preponderance in mortality due to COVID-19 [8,9]. In our study, the male prevalence was undeniable in both waves, which was also statistically significant. Mostly, this is due to hormonal effects and prevalence. Even one of the systematic reviews, which included 32 studies, has shown that mortality among male patients was significantly higher with a pooled odds ratio (OR) and hazard ratio (HR) of 1.49 (95% CI [1.41-1.51]) and 1.24 (95% CI [1.07-1.41]), respectively [11].

Dyspnea has been associated with an increased risk for COVID-19-related deaths in clinical symptoms, OR 3.31 (95%CI [1.78-6.16]) [12]. Even in this study, dyspnea was the most common presenting symptom among the patients in both waves, consistent with the other studies on COVID-19 mortality [13]. Other common symptoms were fever, cough, sore throat, chest pain, altered mental status, abdominal pain, diarrhea, headache, and loss of taste and smell. In one of the studies in Iran, where they compared clinical symptoms in the first and second waves, they found that symptoms like fever, diarrhea, loss of taste and smell, and abdominal pain were more common during the second wave [14]. In this study, we found that cough, altered mental status, headache, and loss of taste and smell were more common symptoms during the second wave, which was statistically significant.

Medical comorbidity is one of the reasons for severe events and higher mortality, as is demonstrated in multiple studies and systemic reviews. In one of the systematic reviews, the serious event is seen in persons with hypertension (OR-2.95, 95% CI [2.21-3.94]) and with diabetes mellitus (OR-3.07, 95% CI [2.02-4.66]) [15]. In another systemic review, hypertension (20.7, 95% CI [13.34-28.88]) was the most prevalent comorbidity, followed by cardiovascular diseases (9.6, 95% CI [4.81-16.23]) and diabetes mellitus (9.55, 95% CI [5.52-17.44]) [16]. Our study found that diabetes was the most common comorbidity among the patients during both COVID-19 waves, with no statistically significant difference. One of the meta-analyses has demonstrated increased mortality and severity of disease in COVID-19 patients with a relative risk (RR) of 2.38 (95% CI, [1.88-3.03]) [17]. Hypertension and cardiovascular disease as comorbidities were seen more during the first wave of COVID-19 than the second wave, which was statistically significant (p< 0.05). Other usual comorbidities seen in both waves were chronic obstructive airway disease, chronic kidney disease, chronic liver disease, malignancy, and chronic neurological disease, mainly cardiovascular accidents.

In this study, lymphopenia was the most common lab parameter in both waves. Lymphopenia at presentation was reported as one of the reasons for a poorer prognosis in COVID-19, as per one of the Korean studies [18]. Another meta-analysis reported that leukocytosis was more prevalent in non-survivors of COVID-19, with a weighted mean difference of 3.66 (95%, CI [2.58-4.74]). They also indicated that the patients who had lymphocytosis, thrombocytopenia, or raised aspartate transaminase had higher mortality [12,19]. A similar study was done at the same center in India, which also showed a raised level of transaminase level during the second wave of COVID-19 [20]. In this study, lymphocytosis, thrombocytopenia, and raised aspartate transaminase were more prevalent during the second wave, resulting in significant deaths. As per one of the meta-analyses, thrombocytopenia increases the risk of severe COVID-19 by over five folds [21,22]. They also found that the patients with severe anemia had more severe disease, with a weighted mean difference of 4.08 (95%, CI [5.12- 3.05]). In this study, also anemia was present in almost 28% of the patients.

Rapid spread and progression of the second wave had caused patients to come to the hospital in more distressing conditions and need more aggressive management and intensive care unit care [23,24]. Most patients during the second wave had tachypnea and hypoxia at presentation. Also, most patients needed an ICU stay and non-invasive ventilation support during their hospital stay. In contrast to the first wave, fewer patients underwent renal replacement therapy during the second wave. This may be attributed to the rapid progression of the disease and short hospital stays [4]. Acute respiratory distress syndrome remains the leading cause of death in COVID-19 patients, as seen in multiple studies and meta-analyses, including the present study [12,25].

Overall, it is observed from a retrospective observational study that age >47 years, associated with comorbidities like hypertension and diabetes, hypoxia, tachycardia, lymphopenia, and raised inflammatory markers, are predictors of in-hospital mortality [26]. A similar thing was observed in the present study in both waves.

Although the majority of the data in both waves of this study is consistent with prior literature, this study has the limitation of a one-center comparison. It would be better if a multicentric comparison was done for the same period of hospitalization. 

## Conclusions

The study was a single-center experience, but the findings correlated with most systematic reviews and meta-analyses. Very few studies worldwide compared patients' baseline characteristics during the first and second COVID-19 waves, including India. The study showed that most of the baseline demographic and clinical parameters attributed to COVID-19 mortality were more common during the second wave and can be one of the possible scientific explanations for the high mortality during the second wave in India. The study highlighted a concerted hospital course during the second wave in terms of ICU, the need for NIV, and inotrope support. There was a significant difference in complications manifesting in the form of ARDS and sepsis, with a higher percentage of complications observed in the second wave. The unpredictable nature of COVID-19 calls for instituting a well-planned surveillance mechanism in place to identify the waves in COVID-19 outbreaks and prompt responses, along with developing infrastructure and capacity to manage complications.
